# Prenatal diagnosis for *de novo* mutations: Experience from a tertiary center over a 10‐year period

**DOI:** 10.1002/mgg3.573

**Published:** 2019-01-28

**Authors:** Ori Eyal, Michal Berkenstadt, Haike Reznik‐Wolf, Hana Poran, Tomer Ziv‐Baran, Lior Greenbaum, Hagit Yonath, Elon Pras

**Affiliations:** ^1^ The Danek Gertner Institute of Human Genetics Sheba Medical Center, Tel Hashomer Ramat Gan Israel; ^2^ Sackler Faculty of Medicine Tel‐Aviv University Tel‐Aviv Israel; ^3^ Sackler Faculty of Medicine School of Public Health Tel‐Aviv University Tel‐Aviv Israel; ^4^ The Joseph Sagol Neuroscience Center Sheba Medical Center, Tel Hashomer Ramat Gan Israel

**Keywords:** de novo, hot spots, mutations, neurofibromatosis Type‐1, prenatal diagnosis, Rett syndrome

## Abstract

**Background:**

This study summarizes the results of prenatal diagnosis due to a history of *de novo* mutation in a previous pregnancy, in a tertiary center in Israel, over a 10‐year period.

**Methods:**

We sorted all cases of *de novo* mutations from a pool of 2,260 pregnancies for which prenatal molecular diagnosis was applied, between the years 2008 and 2017. We identified 122 molecular prenatal diagnosis performed for *de novo* mutations, in 90 women.

**Results:**

While the total number of yearly prenatal diagnoses stayed stable, a linear increase was detected in the number of cases for which the procedure was done due to a previous *de novo* mutation: from 3 cases in 2008 to 24 cases in 2017. The most common diseases were Rett syndrome (19), neurofibromatosis Type‐1 (12) and Tuberous sclerosis (5). Recurrence occurred in 3 of the 90 women (3.3%) and hotspot mutations were identified in two genes accounting for 11 cases. We did not find a difference in paternal age at first occurrence of the *de novo* mutation between the study group and the control group.

**Conclusion:**

The large increase in the annual number of prenatal diagnoses performed due to a previous pregnancy with a *de novo* mutation reflects the growing understanding regarding the role of these mutations in the pathogenesis of genetic diseases.

## 1 INTRODUCTION

Prenatal diagnosis for monogenic diseases is on the rise. Two main factors contribute to this trend. The first is pre‐conception screening programs that intend to identify carriers for autosomal recessive and X‐linked diseases. If both parents are found to be carriers for a recessive disorder or if the female is found to be a carrier for an X‐linked disorder, prenatal diagnosis is offered to the parents. The chance of having an affected baby in these circumstances is 25%. The second is families in which a genetic disease is identified and verified by a molecular analysis. Recurrence rates are usually between 25%–50% depending on the inheritance mode; autosomal recessive, autosomal dominant or X‐linked. A third and less common cause for prenatal diagnosis is a previous diagnosis of a *de novo* mutation. A human zygote inherits half of its genome from the female and the other half from the male. With the genetic information passed on from generation to generation, a small number of novel genetic changes, *de novo* mutations, are formed in each individual. Such changes can occur during the formation of the gametes or postzygotically (Lynch, 2010; Roach et al., 2010). Genome‐wide next generation sequencing studies have estimated the rate of *de novo* single nucleotide variants (SNVs) in humans at 1.0–1.8 × 10^–8^ per nucleotide per generation (Francioli et al., 2015; Gilissen et al., 2014; Goldmann et al., 2016; Michaelson et al., 2012; Rahbari et al., 2016; Roach et al., 2010). This number translates into an average of 60 *de novo* SNVs in the genome of a newly formed embryo, however only one to two of these affect the coding sequence (Francioli et al., 2015; Gilissen et al., 2014; Goldmann et al., 2016; Kong et al., 2012; Michaelson et al., 2012), and only a fraction of those causes genetic diseases. Novel mutations continue to arise throughout postnatal and adult life in both somatic and germ cells. Mutations present in the germ cells can be transmitted to the next generation (Campbell, Shaw, Stankiewicz, & Lupski, 2015). Accordingly, the recurrence rate for a couple that have a child with a genetic disease caused by a *de novo* mutation is higher than that of the general population and is estimated at 1%–4% (Campbell et al., 2014). The single most important known risk factor contributing for *de novo* mutations is advanced paternal age at conception (Kong et al., 2012). Interestingly *de novo* mutations are not equally distributed throughout the human genome and occur more often in genes belonging to RAS–MAPK pathway (Goriely & Wilkie, 2010; Yoon et al., 2013).

In order to prevent recurrence in such families we offer them prenatal diagnosis by amniocentesis between weeks 17–22 of the pregnancy. Here we summarize the results of 90 women who performed prenatal diagnosis due to a history of *de novo* mutation in a previous pregnancy, over a 10‐year period.

## 2 METHODS

### 2.1 Ethical compliance

The study approved by the ethics committee was performed at the Institute of Human Genetics at the Sheba Medical Center, Israel.

### 2.2 Data collection

Information was collected from a pool of medical files from 2,260 women who performed amniocentesis due to an increased risk for a genetic disease in the previous child or fetus. Information retrieved included: genetic diagnosis, gene, mutation, paternal age at the time of conception with the *de novo* mutation pregnancy, outcome of the prenatal diagnosis in following pregnancies focusing on whether the *de novo* mutation had reoccurred. Some families repeated the prenatal diagnoses in more than one pregnancy but were included only once. A control group was obtained from the Israeli Ministry of Health and included the paternal age at the time of conception from 1,248,955 live births, between the years 2008–2015 (data on the years 2016–2017 are not yet available).

### 2.3 Statistical analysis

The statistical analysis was carried out with a one sample *T*‐test.

Sequence references of MECP2 (NG_007107.2), FGFR3 (NG_012632.1), TSC1 (NG_012386.1) and COL1A2 (NG_007405.1) were obtained from GenBank (https://www.ncbi.nlm.nih.gov/genbank/).

## 3 RESULTS

**Figure 1:**
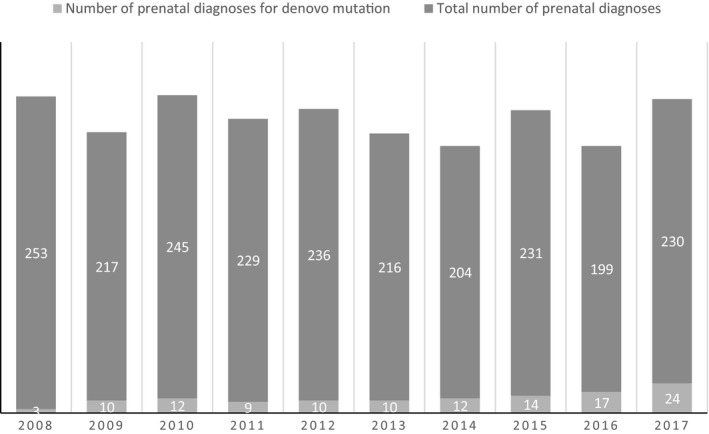
Number of prenatal diagnoses for *de novo* mutations between 2008–2017

Between 2008 and 2017, 122 prenatal diagnosis procedures were performed in 90 pregnancies for a previously identified *de novo* mutation. Thirty‐one women performed the procedure more than once. During this period, we have witnessed an almost 10‐fold increase in the rate of procedures for *de novo* mutations: from 3 in 2007 to 24 in 2017. Interestingly the overall rate of molecular prenatal diagnosis procedures did not increase during this period with an average of 222 procedures per year (Figure 1).

**Figure 2:**
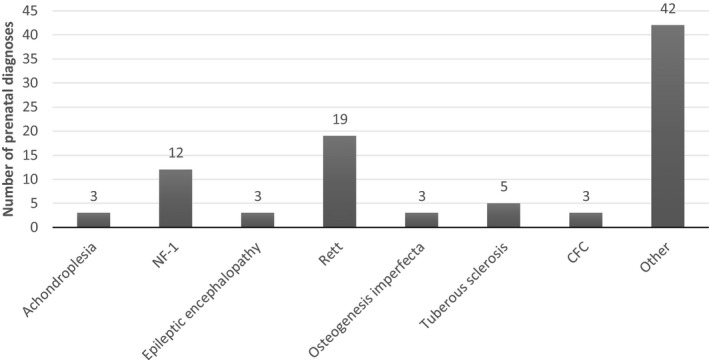
Common *de novo* syndromes

The two most common *de novo* conditions for prenatal diagnosis were Rett syndrome and neurofibromatosis Type‐1, accounting for more than 30% of the cases. Other *de novo* diseases that appeared multiple times are shown in Figure 2. Two mutations were found to occur more than once in our families, representing mutational hotspots: The mutation R168X (c.502 C>T) in *MECP2* (Rett syndrome; OMIM: #300005) repeated itself in eight different families and G380R (c.1138 C>A) in *FGFR3* (Achondroplasia; OMIM: #134934) was found in three different families.

In three families (3.33%), we observed recurrence of the *de novo* mutation in a following pregnancy: a family with osteogenesis imperfecta and a mutation in *COL1A2* (OMIM: #120160), a family with Rett syndrome and a mutation in the *MECP2* and a family with tuberous sclerosis and a mutation in the *TSC1* (OMIM: #605284; Table 1). These cases represent germinal mosaicism in one of the parents and the overall recurrence rate (3.33%) is similar to what has previously been described in the literature (Campbell et al., 2014). Sanger sequencing did not detect low‐level mosaicism of the *de novo* mutations in any of the parents.

**Table 1:** Mutations that reoccurred in the same family

Syndrome	Mutation	No. of recurrences
Osteogenesis imperfecta	*COL1A2‐*G994D (c.2981 G>A)	1 recurrence
Rett	*MECP‐*R294X (c.880 C>T)	1 recurrence
Tuberous sclerosis	*TSC1*‐R786X (c.2356 C>T*)*	1 recurrence

Note

GenBank reference no: COL1A2 (NG_007405.1), MECP2 (NG_007107.2), TSC1 (NG_012386.1).

Surprisingly, we did not find a difference in paternal age at the time of conception of the pregnancy with the *de novo* mutation (32.37 ± 0.3251) and a control group composed of the average paternal age in Israel between the years 2008–2015 in over 1,000,000 fathers (32.82; *p *= 0.629) (Table 2).

**Table 2:** Paternal age in the study group and in the general population

	Average paternal age	Number of cases	*SE*
Study group	32.37	80	0.3251
General population	32.823	1,248,955	

Note

*p* = 0.629.

## 4 DISCUSSION

We have found a steady rise that resulted in an eightfold increase in prenatal diagnosis due to *de novo* mutations, over a 10‐year period. Three explanations could account for this sharp increase. The first is the explosive use of whole exome sequencing (WES) for the identification of genetic diseases that has resulted in a substantial rise in the identification of *de novo* mutations. However as shown in Figure 2, most of the diseases for which prenatal diagnosis were performed were diagnosed on a clinical basis without the need for WES and therefore this explanation by itself cannot be responsible for the increase. The second explanation is growing awareness to the increased recurrence rates due to gonadal mosaicism among medical staff. Improvement in medical services, better education of physicians and nurses, the growing availability and utilization of genetic counseling and prenatal services may explain the large increase in pregnant women with previous offspring affected by *de novo* mutations sent for prenatal diagnosis. The last explanation is a change in referrals characteristics to our center. Over the last decade, new genetic centers have opened, the mixture of cases in our center has changed and the more complex ones were sent to us. Paternal age has been on the rise in the last decades, however in our cohort this explanation cannot account for the increase (Table 2). Taken together the growing number of prenatal diagnoses for *de novo* mutations reflects the growing understanding that such mutations contribute a significant portion of morbidity due to genetic diseases in the general population.

The main reasons for the creation of *de novo* mutations are incorrect incorporation of nucleotides by DNA polymerases ε and δ (Korona, LeCompte, & Pursell, 2010; Schmitt, Matsumoto, & Loeb, 2009; Ségurel, Wyman, & Przeworski, 2014) and by failure of the proofreading subunit present in both polymerases to correct these errors (Ségurel et al., 2014). Their occurrence across the genome is not completely random and factors that play a role in the genome mutability include the local base‐pair context, recombination rate and the timing of replication (Goldmann et al., 2016; Michaelson et al., 2012; Stamatoyannopoulos et al., 2009). Timing of replication refers to the order in which different regions of the genome are replicated during the S‐phase of the cell cycle. On the average those that are replicated late display more newly acquired changes than parts that are replicated early, possibly due to depletion of dNTPs at the end of replication, to alterations in polymerase activity or decreased repair activity (Chen et al., 2010; Koren et al., 2012; Ségurel et al., 2014). Occasionally, multiple *de novo* mutations may occur very close to the other in a given individual, thus creating “mutational clusters.” In addition, *de novo* mutations may appear at the same location in several unrelated individuals thus pointing to the existence of mutational hotspots (Chan & Gordenin, 2015). Mutational hotspots are the probable explanation for the recurrence of the same mutations in our cohort (Table 1).

About 80% of the *de novo* germline point mutations form on the paternal allele, findings that can be explained by the constant division of the spermatogonial cells throughout life resulting in the accumulation of *de novo* mutations. Amazingly, a large increase with paternal age has been observed for a small subset of *de novo* mutations that are highly recurrent and localize to specific nucleotides in the genome. Some investigators have hypothesized that spermatogonial stem cells with mutations in genes in the RAS–MAPK pathway exert a growth advantage that may lead to their clonal expansion in the testis (Goriely & Wilkie, 2010; Yoon et al., 2013). Such positively selected mutations offer sperm cells a selective advantage in the testis despite being detrimental at the organism level and have been described in a host of genetic diseases including Apert, Crouzon, and Pfeiffer syndromes (Goriely, McVean, Röjmyr, Ingemarsson, & Wilkie, 2003; Maher et al., 2016), Noonan, and Costello syndromes (Goriely et al., 2009; Maher et al., 2016; Yoon et al., 2013), Muenke syndrome, achondroplasia and thanatophoric dysplasia (Goriely et al., 2009; Maher et al., 2016), and multiple endocrine neoplasia (Choi, Yoon, Calabrese, & Arnheim, 2012). Indeed 22% of the *de novo* mutations that we have found are in genes that belong to the RAS–MAPK (Table 3).

**Table 3:** RAS‐MAP kinase associated diseases

Genetic disease	Number of cases
Achondroplasia	3
Cardio‐facio‐cutaneous	3
Neurofibromatosis Type‐1	12
PTEN	1
Thanatophoric dysplasia	1

In 3 of the 90 pregnancies (3%), we have detected recurrence of the *de novo* mutation, indicating germline mosaics in one of the parents (Table 1). These figures are compatible with previous reports in the medical literature (Campbell et al., 2014).

*de novo* mutations can be further delineated to include two additional subgroups: approximately 4% originate from parental mosaicism detectable in blood samples of one of the parents (Rahbari et al., 2016), for which recurrence risk is higher and has been estimated at above 5% (Campbell et al., 2014). In contrast, postzygotic events in the embryo account for 7% of what may initially appear as *de novo* mutations (Acuna‐Hidalgo et al., 2015; Besenbacher et al., 2015; Dal et al., 2014) and for these recurrence risks in following pregnancies are similar to the general population (Biesecker & Spinner, 2013). Differentiating these two subgroups by targeted deep sequencing of blood samples from the parents and affected offspring may provide a personalized and more accurate estimate of the recurrence risk (Acuna‐Hidalgo, Veltman, & Hoischen, 2016).

## CONFLICT OF INTEREST

The authors declare no conflict of interest.

## AUTHOR CONTRIBUTION

Conceptualization: E.P. H.Y.; Methodology: O.E., M.B., H.R.W., H.P., E.P.; Investigation: O.E., M.B., H.R.W., L.G., H.P., T.Z.B., E.P.; Writing: O.E., L.G., T.Z.B., E.P.; Supervision H.Y., E.P.; Manuscript editing and revision for intellectual content: all authors.
